# Selective serotonin reuptake inhibitors and risk of epilepsy after traumatic brain injury – A population based cohort study

**DOI:** 10.1371/journal.pone.0219137

**Published:** 2019-07-19

**Authors:** Jakob Christensen, Henrik Schou Pedersen, Morten Fenger-Grøn, Jesse R. Fann, Nigel C. Jones, Mogens Vestergaard

**Affiliations:** 1 Department of Neurology, Aarhus University Hospital, Aarhus, Denmark; 2 Research Unit for General Practice and Section for General Medical Practice, Department of Public Health, Aarhus University, Aarhus, Denmark; 3 Department of Psychiatry, University of Washington, Seattle, Washington, United States of America; 4 Department of Medicine (RMH), University of Melbourne, Melbourne Brain Centre, Parkville, Victoria, Australia; 5 Department of Neuroscience, Central Clinical School, Monash University and Department of Neurology, The Alfred Hospital, Melbourne, Victoria, Australia; Hunter Holmes McGuire VA Medical Center, UNITED STATES

## Abstract

**Objective:**

Traumatic brain injury (TBI) is common and associated with a marked increased risk of developing epilepsy. Animal studies indicate that treatment with selective serotonin reuptake inhibitors (SSRIs) may increase the risk of epilepsy after TBI. The aim of this study was to investigate whether use of SSRIs modifies the risk of epilepsy after TBI.

**Methods:**

This was a cohort study of 205,715 persons, who suffered a TBI in Denmark from 1996 to 2013. For each person with TBI, we matched 10 reference persons (N = 2,057,150) who were alive on the day of TBI and who had the same age and gender but had no history of TBI. We used a stratified Cox regression to calculate the relative risk of epilepsy after TBI for persons exposed to TBI, SSRI or both after adjustment for income, civil status, medical and neurological comorbidities, severe mental disease, and substance abuse.

**Results:**

The risk of epilepsy was 5.61 times higher for persons who used SSRI at time of TBI (adjusted Hazard Ratio (aHR): 5.61 (95% CI: 4.88; 6.45)), 3.23 times higher for persons who had a TBI but did not use SSRI at time of TBI (aHR: 3.23 (95% CI: 3.12;3.35)), and 1.31 times higher for persons who used SSRI but had no TBI (aHR: 1.31 (95% CI: 1.18; 1.45)) compared to persons unexposed to both TBI and SSRI.

**Conclusions:**

This large population based cohort study showed that people using SSRI at the time of a TBI had higher risk of developing epilepsy compared to people not using SSRI at the time of TBI. The results are in line with those of animal studies and calls for further studies to evaluate whether the association is due to SSRIs or to the underlying disease (e.g. depression or anxiety).

## Introduction

Traumatic brain injury (TBI) is an important cause of death and hospital admissions worldwide. There were approximately 2.8 million TBI-related emergency department visits, hospitalizations, and deaths in the United States in 2013[1, 2], and more than 1.4 million hospital admission and deaths related to TBI in Europe in 2012.[3] The consequences of TBI as measured by age-standardized disability-adjusted life-years lost are significant and the Global Burden of Disease Study 2016 identified TBI as a leading cause of disability worldwide.[4]

Among the consequences of TBI, post-traumatic epilepsy is a major concern and cumulative incidence of epilepsy after TBI increased from 1.0% in one year to 4.0% in nine years after TBI in a US study.[5] The risk of epilepsy after TBI is closely linked to the severity of TBI.[6, 7] The cumulative incidence of post traumatic epilepsy in the first 3 years after discharge, after adjusting for loss to follow-up, was 4.4 per 100 persons over 3 years for hospitalized mild TBI, 7.6 for moderate TBI, and 13.6 for severe TBI.[8, 9]The time from brain injury to epilepsy onset may span several years,[6] leaving room for clinical intervention that may prevent the subsequent development of epilepsy (i.e. epileptogenesis).[10] Preventing epilepsy and improving pre-clinical models that adequately represent posttraumatic epilepsy is critical for enhancing our understanding of injury progression.[9] However, little is known about factors that may modify the risk of epilepsy after TBI in humans and several human clinical trials have not yet been successful in preventing epilepsy in persons exposed to TBI.[11].[12]

Animal models suggest that changes in glutamate and GABA signaling may play a role in acute seizure onset following TBI.[9] However, it is not clear why some recover from these transient signal changes while others go on to develop epilepsy after TBI.[9] Several attempts have been made in order to prevent epileptogenesis in animals–e.g. studies suggest that treatment with antiepileptic drugs after TBI may decrease the risk of epilepsy,[10, 12], but this has not been replicated in humans.[11, 12] Selective serotonin reuptake inhibitors (SSRIs) appear to be safe to use in chronic epilepsy, and possibly have anticonvulsant properties in established epilepsy,[13] this anticonvulsant effect appears to be separate to the pro-epileptogenic effects. Recently, animal studies indicated that treatment with SSRIs enhanced the rate of epileptogenesis, thereby potentially increasing the risk of epilepsy after brain insults including TBI.[14–16] The mechanism underlying the pro-epileptogenic potential of SSRIs in animals is unknown, but this effect is not mediated by 5-HT2A receptors. [16]Long-term treatment with SSRI in humans is common, but no studies in humans have yet evaluated whether the use of SSRIs and other antidepressants at time of TBI modifies the risk of epilepsy after TBI.

We studied whether the use of SSRIs and other antidepressants at time of TBI influenced the risk of epilepsy after TBI in humans by using a nationwide cohort study in Denmark.

## Materials and methods

### Study design and setting

This is a matched cohort study of all persons who suffered a TBI treated as outpatients in emergency rooms or as inpatients at hospitals in Denmark from 1 January 1996 to 31 December 2013. For each person with TBI, we matched 10 reference persons who were alive at the day of TBI and who had the same age and gender.

### Danish health registers

Data were retrieved from several Danish registers and linked through the Danish civil registration number (CPR number) from the Danish Civil Registration System.[17] The Danish civil registration number is a unique identification number given to each citizen living in Denmark ensuring accurate linkage between registers.

### Exposure to TBI

Information on TBIs was obtained from the Danish National Patient Register which contains information on all medical hospital admissions in Denmark since 1977 and outpatient medical and emergency room contacts since 1995.[18] The International Classification of Diseases, Eighth Revision (ICD-8)[19] was the diagnostic instrument used in Denmark up to January 1, 1994 when it was replaced by the International Statistical Classification of Diseases, 10th Revision (ICD-10).[20] All inpatient contacts with a primary discharge diagnosis of TBI and all outpatient contacts to an emergency room with a diagnosis of TBI between 1996 and 2013 were identified. We used the ICD classification of TBI to describe TBI severity based on a previous population-based study of seizures after traumatic brain injuries.[7] In this study by Annegers et al., TBI were classified into “mild (loss of consciousness or amnesia lasting less than 30 minutes), moderate (loss of consciousness for 30 minutes to 24 hours or a skull fracture), or severe (loss of consciousness or amnesia for more than 24 hours, subdural hematoma, or brain contusion)” (for diagnostic ICD codes please see S1 Table). The definition of mild TBI (concussion) in Denmark is based on the definition given by the American Congress of Rehabilitation Medicine. [21] The diagnostic criteria include a relevant direct trauma to the head manifesting with changed brain function (i.e., loss of consciousness, amnesia, confusion/disorientation, or focal [temporary] neurological deficit). Severity of mild TBI should not include loss of consciousness longer than 30 min, a Glasgow Coma Scale (GCS) of 13 or less after 30 min, or post-traumatic amnesia longer than 24 hours. Thus, GCS is not the only criteria for mild TBI and although GCS is not registered in the Danish National Hospital Register, the GCS is included among the diagnostic criteria. Severe TBI (structural brain injury) includes brain contusion or intracranial haemorrhage. Although GCS was not registered for this group of persons with TBI, the consequences of the injury clearly exceeds the consequences of the mild TBI. The ability to classify an intermediate category of “Moderate TBI” characterized by length of amnesia and GCS would have been interesting, but it was not possible to retrieve this information from the registers. In order to include all individuals with TBI, we also included a category “skull fracture” which indicates an injury to the head of some severity, although again the diagnostic criteria do not include length of amnesia and GCS and the severity cannot be categorized precisely. For each contact, the TBI was categorized according to a hierarchy of brain injury severity—severe brain injury, skull fracture, and mild brain injury. In addition, mild TBI was characterized as either mild TBI without a hospitalization (i.e., emergency contacts only) or mild TBI with hospitalization. Contacts for brain injuries recorded in the same patient within 14 days were categorized as the same event.

### Exposure to SSRI

The Danish National Prescription Registry holds information on all redeemed prescriptions since 1 January 1996.[22] Taking SSRIs in Denmark require a prescription but drugs used only in hospitals are not captured in the Danish National Prescription Registry. We included information on all redeemed prescriptions for SSRIs (ATC code: N06AB) from 1 January 1996 to 31 December 2013. We categorized persons as exposed to SSRIs if they redeemed at least one prescription for SSRIs between 0 and 3 months before the day of the TBI (or index date if this was a reference person). We did not include information on exposure to SSRIs after time of TBI to avoid confounding from SSRI used in the treatment of symptoms stemming from the TBI.

### Outcome

We identified persons diagnosed with epilepsy in the Danish National Patient Register (ICD8 code: 345 and ICD10 codes: G40 and G41). We excluded all persons with an epilepsy diagnosis prior to date of TBI.

### Covariates

We used the household income data from Statistics Denmark the year before the TBI (index date) (divided into quintile). For persons who were between 0 and 24 years of age at time of TBI (index date), we used the parental income (or the highest parental income if the parents were not living together). We obtained information from the Danish Civil Registration System on civil status between 1996 and 2013, dichotomized into living with a partner (i.e. marriage, registered partnership or cohabitation) or living alone (i.e. living without a partner, including widows and widowers).

Diagnoses of psychiatric disorders are entered in the Danish Psychiatric Central Research Register,[23] which includes information on all inpatient admissions to psychiatric hospitals and psychiatric wards in general hospitals in Denmark since April 1, 1969—outpatient contacts have been included since January 1, 1995. We obtained information on substance abuse (excluding tobacco abuse) between 1969 and 2013 from the Danish Psychiatric Central Research Register or from the Danish National Patient Register (S2 Table).

Information on medical and neurological comorbidities was obtained between 1977 and 2013 from the Danish National Patient Register. We obtained information on comorbid medical and neurological conditions, including ischemic heart disease, congestive heart failure, atrial fibrillation or flutter, peripheral vascular disease, cerebrovascular disease, autoimmune disease, and HIV (S2 Table).

### Sensitivity analyses

First, we analyzed the risk of epilepsy when restricting to persons born after 1977 (i.e. persons with complete follow up from birth in the Danish National Hospital Register).

Secondly, we analyzed the risk of epilepsy after TBI in persons exposed to other antidepressants at time of TBI (tricyclic antidepressants (TCAs); ATC code: N06AA, monoamine oxidase, inhibitors (MAOIs); ATC code: N06AF, N06AG and other antidepressants (ATC Code: N06AX)). In order to be able to compare the individual antidepressants, the reference group was restricted to the cohort who did not reimburse any kind of antidepressants in the 3 months leading up to the TBI of the index person (index date). There were few users of MAOIs and we therefore grouped the users of MAOIs with the users of antidepressants in poly-therapy (i.e. persons who reimbursed prescriptions for more than one type of antidepressants from 0 to 3 months prior to TBI (index date)). Bupropion is an SSRI which may lower seizure threshold.[24] There were only 412 persons in the entire cohort who reimbursed a prescription for bupropion in the three months leading up to the index date, and among these there were too few cases (<5) with TBI and epilepsy to allow separate analyses.

Thirdly, we compared the risk of epilepsy after TBI in SSRI exposed persons with the risk of epilepsy after TBI in previously SSRI exposed persons. In this analysis, we chose the exposure window from 6 months prior to TBI as opposed to 3 months used in the other analyses, in order to be reasonably sure that the persons did not use SSRIs at time of TBI. Thus, SSRI exposed persons were those who redeemed prescriptions for antidepressants from 6 months before to the day of TBI (index date) and previously SSRI exposed persons were those who redeemed prescriptions for antidepressants from 12 months to 6 months prior to day of TBI (index date)), but not in the 6 months leading up to the TBI (index date).

Fourthly, we analyzed the risk of epilepsy by use of SSRIs at time of TBI excluding persons with concomitant use of antiepileptic (ATC code NO3A) and psycholeptic drugs (ATC code N05A, N05B and N05C).

Fifthly, we analyzed the risk of epilepsy after TBI stratified on hospital admission with depression registered in the Danish Psychiatric Central Research Register (ICD 10 codes F32 –F33) and use of SSRIs at time of TBI.

### Statistical methods

Individuals were followed from date of TBI of the index person (index date) until date of epilepsy, date of death, December 31 2013, or date of emigration whichever came first. This analysis was done using a stratified Cox regression, allowing each matched stratum to have its own baseline hazard function. Estimates were adjusted for baseline values (income, civil status, medical and neurological comorbidities, schizophrenia, bipolar affective disorder, and substance abuse).

The proportional hazards assumption was assessed graphically on all variables using log-minus-log plots. We used two-sided significance tests for all analyses with statistical significance set at P < 0.05. Analyses were performed using STATA 13 (Stata Corporation, College Station, TX).

The study was approved by the Danish Data Protection Agency.

## Results

### Participants

Characteristics of study participants are shown in Table 1. People with TBI more often used SSRIs, had lower income, were more often single and registered with severe mental illness, drug and alcohol abuse, and severe medical conditions than people without TBI.

**Table 1:** Characteristics of patients with traumatic brain injury and their reference persons^a^.

	Traumatic Brain Injury		No Traumatic Brain Injury		Total
	Number	Percent	Number	Percent	Number
Total population	205,715	100.00%	2,057,150	100.00%	2,262,865
					
Selective serotonin reuptake inhibitors					
No	194,131	94.37%	2,009,626	97.69%	2,203,757
Yes	11,584	5.63%	47,524	2.31%	59,108
					
Income (quantile)^b^					
<118.000 DKK/year	47,426	23.05%	409,568	19.91%	456,994
118.000–155.000 DKK/year	44,301	21.54%	409,560	19.91%	453,861
155.000–193.000 DKK/year	40,476	19.68%	409,570	19.91%	450,046
193.000–252.000 DKK/year	37,467	18.21%	409,559	19.91%	447,026
>252.000 DKK/year	35,479	17.25%	409,561	19.91%	445,040
Missing	566	0.28%	9,332	0.45%	9898
					
Marital status					
Single	145,598	70.78%	1,323,198	64.32%	1,468,796
Married	44,008	21.39%	559,293	27.19%	603,301
Cohabitant	16,109	7.83%	174,659	8.49%	190,768
					
Severe mental illness					
No	203,776	99.06%	2,047,387	99.53%	2,251,163
Yes	1939	0.94%	9,763	0.47%	11,702
					
Alcohol abuse					
No	190,012	92.37%	2,018,751	98.13%	2,208,763
Yes	15,703	7.63%	38,399	1.87%	54,102
					
Drug abuse					
No	201,745	98.07%	2,047,344	99.52%	2,249,089
Yes	3970	1.93%	9,806	0.48%	13,776
					
Ischemic heart disease					
No	196,356	95.45%	1,983,685	96.43%	2,180,041
Yes	9359	4.55%	73,465	3.57%	82,824
					
Chronic heart failure					
No	205,003	99.65%	2,052,152	99.76%	2,257,155
Yes	712	0.35%	4,998	0.24%	5710
					
Atrial fibrillation					
No	200,120	97.28%	2,020,329	98.21%	2,220,449
Yes	5595	2.72%	36,821	1.79%	42,416
					
Peripheral vascular disease					
No	202,569	98.47%	2,032,099	98.78%	2,234,668
Yes	3146	1.53%	25,051	1.22%	28,197
					
Cerebrovascular disease					
No	196,245	95.40%	2,006,436	97.53%	2,202,681
Yes	9470	4.60%	50,714	2.47%	60,184
					
Autoimmune disease					
No	197,739	96.12%	1,994,133	96.94%	2,191,872
Yes	7976	3.88%	63,017	3.06%	70,993
					
Human immunodeficiency virus (HIV)					
No	205,576	99.93%	2,056,386	99.96%	2,261,962
Yes	139	0.07%	764	0.04%	903

^a^For each person with traumatic brain injury we matched 10 persons with the same sex, age, index date (time of traumatic brain injury) and calendar time.

^b^DKK/year; Danish Kroner per year, 100 Danish Kroner is equivalent to 13.5 € and 16.0 US$

### Main results

Overall, TBI was associated with a threefold increase in risk of epilepsy (aHR: 3.31 (95% CI: 3.19;3.43) (Table 2)). The risk of epilepsy increased with the severity of the TBI; for example, the risk was almost tenfold higher in persons with severe TBI compared to persons without TBI (aHR: 9.91 (95% CI: 9.12; 10.76)) (Table 2).

**Table 2:** Risk of epilepsy by severity of traumatic brain injury.

				Risk of Epilepsy	
Overall risk of epilepsy	Total (Number)	Epilepsy (Number)	Person Years	Crude (95% CI)	Adjusted^a^ (95% CI)
Traumatic brain injury	205,715	4,858	1,698,326	3.83 (3.71;3.96)	3.31 (3.19;3.43)
No traumatic brain injury	2,057,150	13,895	17,881,711	1.00 (ref)	1.00 (ref)
**Risk of epilepsy by traumatic brain injury severity**					
Mild traumatic brain injury (ER only)	81,663	1,150	661,249	2.45 (2.30;2.62)	2.22 (2.08;2.38)
No mild traumatic brain injury (ER only)	816,630	4,851	6,747,844	1.00 (ref)	1.00 (ref)
Mild traumatic brain injury (hospitalized)	94,996	2,108	862,313	3.30 (3.14;3.46)	2.82 (2.67;2.97)
No traumatic brain injury (hospitalized)	949,960	6,957	8,994,509	1.00 (ref)	1.00 (ref)
Skull fracture	5,701	153	46,934	4.48 (3.69;5.44)	4.00 (3.28;4.88)
No skull fracture	57,010	378	495,856	1.00 (ref)	1.00 (ref)
Severe traumatic brain injury	23,355	1,447	127,831	11.94 (11.03;12.92)	9.91 (9.12;10.76)
No severe traumatic brain injury	233,550	1,709	1643,501	1.00 (ref)	1.00 (ref)

^a^Adjusted for civil status, income, medical and neurological comorbidities, schizophrenia, bipolar affective disorder, and substance abuse.

ER only: Emergency Room only

Considering exposure to SSRI, we found that the risk of epilepsy after TBI was five times higher in persons who used SSRI at the time of TBI (aHR: 5.61 (95% CI: 4.88; 6.45)) and three times higher (aHR: 3.23 (95% CI: 3.12; 3.35)) in persons who did not use SSRI at time of TBI when compared to persons who were exposed to neither TBI nor SSRI (Table 3). In persons without TBI, redemption of a prescription for SSRIs was associated with a 1.31 times increased risk of epilepsy (aHR: 1.31 (95% CI: 1.18; 1.45)) (Table 3). Thus, the risk of epilepsy associated with the combination of TBI and SSRI use at time of injury exceeds the sum of the individual risks associated with TBI and SSRI use (i.e. 3.23 + 1.31–1 < 5.61) (Table 3).

**Table 3:** Risk of epilepsy by use of Selective Serotonin Reuptake Inhibitors (SSRIs) at time of traumatic brain injury.

					Risk of Epilepsy		
All traumatic brain injury		Total (number)	Epilepsy (number)	Person Years	Crude (95% CI)	Adjusted^a^ (95% CI)	Adjusted^a^ (95% CI)
Traumatic brain injury	SSRI	11,584	453	61,099	9.17 (8.04;10.45)	5.61 (4.88;6.45)	1.74 (1.50; 2.00)
	No SSRI	194,131	4,405	1,637,228	3.67 (3.55;3.81)	3.23 (3.12;3.35)	1.00 (ref)
No traumatic brain injury	SSRI	47,524	495	256,722	1.80 (1.63;1.99)	1.31 (1.18;1.45)	1.31 (1.18;1.45)
	No SSRI	2,009,626	13,400	17,624,989	1.00 (ref)	1.00 (ref)	1.00 (ref)
					
**Mild traumatic brain injury (ER only)**							
Mild traumatic brain injury (ER only)	SSRI	3,518	112	19,243	8.59 (6.65;11.11)	5.56 (4.23;7.30)	2.61 (1.97; 3.46)
	No SSRI	78,145	1,038	642,006	2.31 (2.15;2.47)	2.13 (1.98;2.28)	1.00 (ref)
No mild traumatic brain injury (ER only)	SSRI	14,979	134	82,308	1.79 (1.47;2.17)	1.37 (1.12;1.68)	1.37 (1.12;1.68)
	No SSRI	801,651	4,717	6,665,536	1.00 (ref)	1.00 (ref)	1.00 (ref)
					
**Mild Traumatic brain injury (hospitalized)**							
Mild Traumatic brain injury (hospitalized)	SSRI	5,625	186	33,574	6.64 (5.49;8.03)	3.92 (3.20;4.80)	1.40 (1.14; 1.73)
	No SSRI	89,371	1,922	828,739	3.20 (3.04;3.37)	2.80 (2.65;2.95)	1.00 (ref)
No mild Traumatic brain injury (hospitalized)	SSRI	21,545	252	126,516	2.03 (1.77;2.34)	1.46 (1.26;1.69)	1.46 (1.26;1.69)
	No SSRI	928,415	6,705	8,867,994	1.00 (ref)	1.00 (ref)	1.00 (ref)
					
**Skull fracture**							
Skull fracture	SSRI	273	8	1475	5.56 (2.29;13.50)	3.57 (1.42;8.99)	0.87 (0.34; 2.22)
	No SSRI	5,428	145	45,459	4.57 (3.75;5.58)	4.13 (3.36;5.06)	1.00 (ref)
No skull fracture	SSRI	1,276	18	7,004	2.73 (1.60;4.65)	1.92 (1.11;3.34)	1.92 (1.11;3.34)
	No SSRI	55,734	360	488,853	1.00 (ref)	1.00 (ref)	1.00 (ref)
					
**Severe traumatic brain injury**							
Severe traumatic brain injury	SSRI	2,168	147	6807	21.54 (16.00;28.98)	13.44 (9.83;18.38)	1.38 (1.00; 1.91)
	No SSRI	21,187	1,300	121,025	11.59 (10.67;12.59)	9.72 (8.92;10.60)	1.00 (ref)
No severe traumatic brain injury	SSRI	9,724	91	40,895	1.57 (1.25;1.97)	1.10 (0.87;1.39)	1.10 (0.87;1.39)
	No SSRI	223,826	1,618	1,602,606	1.00 (ref)	1.00 (ref)	1.00 (ref)

^a^ Adjusted for civil status, income, medical and neurological comorbidities, schizophrenia, bipolar affective disorder, and substance abuse.

A higher risk of epilepsy after TBI in persons who used SSRIs was found regardless of TBI severity although the super additive effects of TBI and SSRI was not found in persons with skull fracture (Table 3).

**Fig 1:**
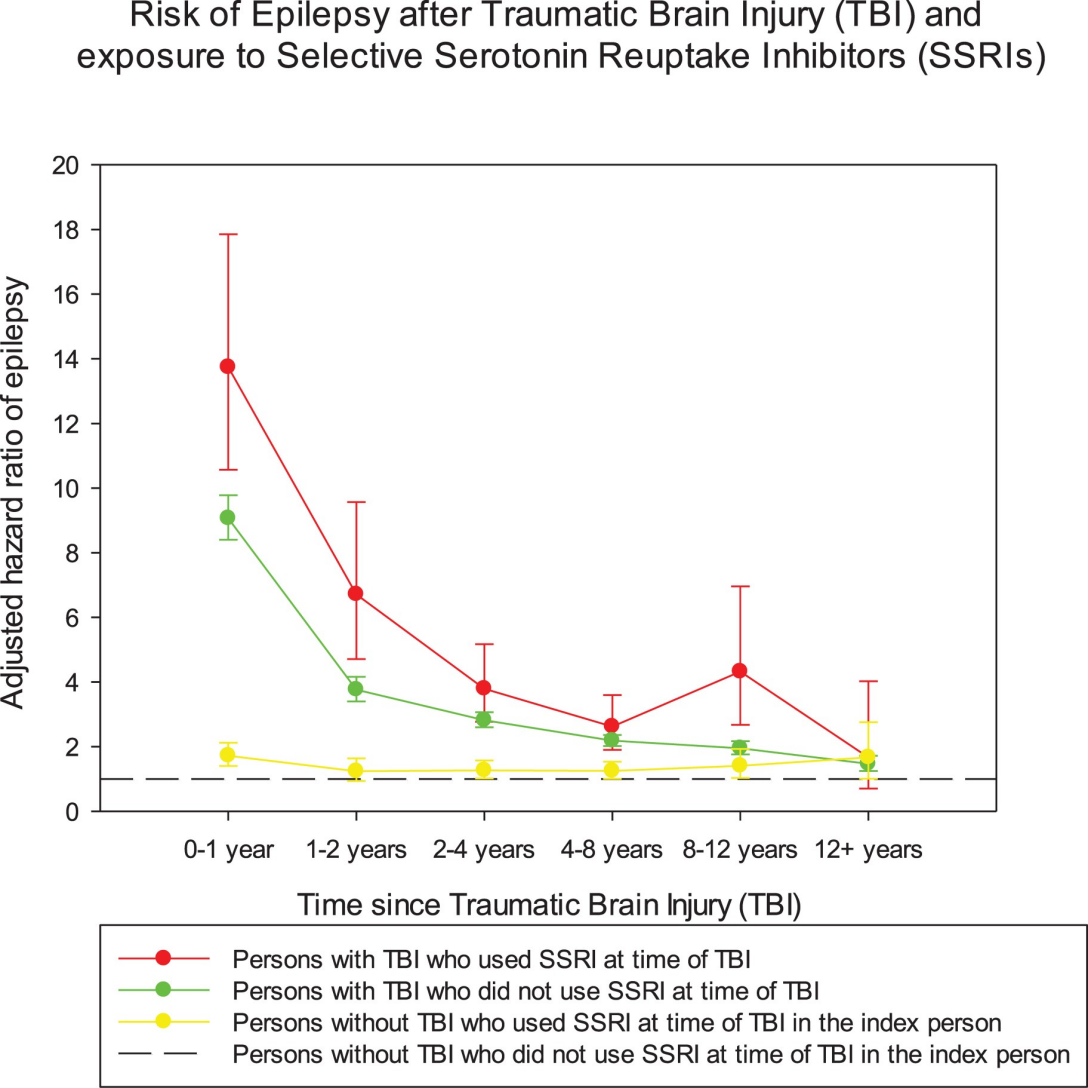
Risk of epilepsy after traumatic brain injury (TBI) and use of Selective Serotonin Reuptake Inhibitors (SSRIs) at time of injury.

The risk of epilepsy was especially high shortly after the TBI and then decreased with time (Fig 1). Persons who used SSRI at time of TBI had the highest risk of epilepsy throughout the follow up period of more than 10 years after the TBI, but the differences between the three categories decreased with time since TBI (Fig 1).

### Sensitivity analyses

After restricting the analyses to persons born after 1 January 1977, the risks of epilepsy associated with TBI and SSRI use at time of injury remained unchanged (S3 Table).

The risk of epilepsy for persons exposed to both TBI and antidepressants varied with the type of antidepressants (S4 Table). The aHR of epilepsy after TBI was 5.62 (95% CI: 4.84; 6.52) for persons taking SSRIs in monotherapy, 3.00 (95% CI: 2.01; 4.47) for persons taking TCA in monotherapy, 8.12 (95% CI: 6.01; 10.98) for persons taking ‘other antidepressants’ in monotherapy and 7.15 (95% CI: 4.91;10.42) for persons taking MAOIs or using antidepressants in poly-therapy when compared to persons not taking antidepressants. The super additive effect was found for all antidepressants except for TCA (S4 Table)

The risk of epilepsy decreased with time since TBI. We therefore restricted the population to persons and reference persons with TBI from 1 Jan 1996 to 31 December 2009 who were then followed up to a maximum of 4 years after TBI (index date) thereby ensuring that follow up was equal for all persons. In this restricted analysis, the risk of epilepsy was 7.43 times higher for persons who used SSRI at time of TBI (Hazard Ratio (aHR): 7.43 (95% CI: 6.13;9.00)), 4.51 times higher for persons who had a TBI but did not use SSRI at time of TBI (aHR: 4.51 (95% CI: 4.28;5.75))), and 1.32 times higher for persons who used SSRI but had no TBI (aHR: 1.32 (95% CI: 1.14;1.53)) compared to persons unexposed to both TBI and SSRI. (S5 Table)

The risk of epilepsy after TBI in people who used SSRIs at time of injury tended to be higher for persons who redeemed a prescription 6–12 months before TBI, but did not redeem prescriptions from 6 months before the day of TBI (aHR: 7.53 (95% CI: 5.39;10.51)) when compared with the risk in persons who redeemed prescriptions also in the 6 months leading up to the TBI (aHR: 5.33 (95% CI: 4.57;6.22)) (Table 4).

**Table 4:** Risk of epilepsy by time of Selective Serotonin Reuptake Inhibitor (SSRI) redemption prior to traumatic brain injury (TBI).

	SSRI redemption 6–12 months prior to day of TBI	SSRI redemption 6 months prior to day of TBI	Total (number)	Epilepsy (number)	Person Years	Risk of Epilepsy		
						Crude (95% CI)	Adjusted^a^ (95% CI)	Adjusted^a^ (95% CI)
					
Traumatic brain injury	Yes	No	1883	85	11,799	11.25 (8.21;15.41)	7.53 (5.39;10.51)	2.35 (1.68; 3.28)
	No	Yes	3961	177	23,423	10.03 (8.09;12.44)	6.08 (4.84;7.64)	1.90 (1.51; 2.40)
	Yes	Yes	9760	360	49,480	8.85 (7.66;10.23)	5.33 (4.57;6.22)	1.66 (1.42; 1.95)
	No	No	190,111	4236	1,613,624	3.61 (3.49;3.74)	3.20 (3.09;3.32)	1.00 (ref)
No traumatic brain injury	Yes	No	10,227	109	67,916	1.78 (1.45;2.18)	1.39 (1.12;1.72)	1.39 (1.12;1.72)
	No	Yes	13,348	157	86,508	1.88 (1.58;2.23)	1.49 (1.24;1.77)	1.49 (1.24;1.77)
	Yes	Yes	44,465	455	231,097	1.88 (1.69;2.08)	1.34 (1.21;1.50)	1.34 (1.21;1.50)
	No	No	1,989,110	13,174	17,496,190	1.00 (ref)	1.00 (ref)	1.00 (ref)

^a^Adjusted for civil status, income, medical and neurological comorbidities, schizophrenia, bipolar affective disorder, and substance abuse.

The aHR of epilepsy after exposure to TBI and SSRI was 5.80 (95% CI: 5.00; 6.74) when excluding persons exposed to antiepileptic drugs, 5.73 (95% CI: 4.84; 6.79) when excluding persons exposed to antipsychotic drugs, 5.93 (95% CI: 5.03; 7.00) when excluding persons exposed to anxiolytic drugs and 5.40 (95% CI: 4.63; 6.30) when excluding persons using hypnotic and sedative drugs when compared to persons without TBI not exposed to SSRI (S6 Table)

The risk of epilepsy after TBI and SSRI exposure stratified on a hospital admission with a diagnosis of depression at any time prior to TBI is shown in S7 Table. The aHR of epilepsy among persons with TBI admitted with depression and exposed to SSRI was 4.84 (95% CI:3.41;6.86), and the aHR of epilepsy among persons with TBI admitted with depression who were not exposed to SSRI was 5.76 (95% CI:4.56;7.28) when compared to persons without TBI who were not admitted with depression and who were not exposed to SSRI. The aHR of epilepsy among persons with TBI who were not admitted with a depression, but were exposed to SSRI was 5.88 (95% CI:5.05;6.84) and the aHR of epilepsy among person with TBI who were not admitted with a depression and not exposed to SSRI was 3.20 (95% CI:3.09;3.32), when compared to persons without TBI who were not admitted with depression and who were not exposed to SSRI (S7 Table)

## Discussion

In this large nationwide cohort study, TBI was associated with higher risk of epilepsy in persons who used SSRIs at the time of the TBI compared to those who did not use SSRIs, suggesting a facilitating effect of SSRI exposure on the development of epilepsy after TBI. Our study was inspired by recent animal studies suggesting that SSRIs influenced the neurobiological mechanisms implicated in epileptogenesis. [14–16] However, it is important to stress that the evidence for a modulating effect of SSRI on development of epilepsy after TBI remains sparse; our study is–as far as we know—the first to present the association in humans.[25] Further, given the co-morbidity between epilepsy and psychiatric disorders and possible bidirectionality between these disorders,[26–28] it is inherently hard to differentiate the effects of SSRI from the effects of the underlying disorders as discussed below.

Whereas tricyclic antidepressants (TCAs) have long been recognized to trigger seizures in persons without epilepsy, growing evidence suggests that newer antidepressants such as SSRIs may potentially lead to reductions in seizure frequency and severity in persons with epilepsy,[15, 29–31] although most studies on the effects on seizures have been descriptive.[32–34] Few studies have evaluated whether antidepressants may influence the processes associated with development of epilepsy after an insult (i.e. epileptogenesis). [25, 35] A recent propensity score matched population based register study from Taiwan found that treatment with SSRIs after stroke was associated with a more than twofold risk of epilepsy (aHR: 2.45 95% CI, 1.69–3.57) compared with persons that were not treated with SSRIs.[35] However, as in the present study, confounding from underlying disorders may confound this association.[30]

Randomized studies have assessed the effects of SSRIs on the risk of developing depression after TBI, [36–39] these studies were unfortunately underpowered to study the modifying effect of SSRI on the occurrence of seizures after TBI. [37, 39] Randomized studies of antidepressants in persons with primary depressive disorders without TBI have found that the incidence of seizures was significantly lower among patients assigned to antidepressants compared to placebo.[31] However, the neurobiological mechanisms underlying development of seizures in persons with primary depressive disorders may differ from the underlying neurobiological mechanisms implicated in epileptogenesis following TBI. [14–16]

In this study, SSRIs was associated with increase in the risk of post traumatic epilepsy but as mentioned the associations found may be confounded by the underlying condition for the SSRI treatment. A bidirectional relationship has been identified between epilepsy and depression in particular.[27, 40, 41] It has been suggested that common underlying pathophysiological mechanisms may explain the risk of developing epilepsy following onset of depression.[26] In this study, the risk of epilepsy after TBI in persons who used SSRIs 6–12 months before TBI, but not in the 6 months leading up to the TBI had the same magnitude and direction as the risk in persons also using SSRIs in the 6 months leading up to the TBI. This suggests that it may not be the use of SSRIs at time of injury or a continuous effect of SSRI on the brain that are responsible for the association, but possible also the underlying condition or other unmeasured confounding (Table 4). However, we adjusted for socioeconomic status, substance abuse and severe mental disorders all of which are associated with TBI and may possibly increase the risk of epilepsy.

The risk of epilepsy after TBI was increased for all users of antidepressants except for users of TCAs (S4 Table). This may suggest that the mechanism behind the increased risk of epilepsy after TBI in persons exposed to these drugs is similar–alternatively that the underlying condition may be accountable for the association. TCAs have other indications than SSRIs (e.g. neuropathic pain) and other studies have suggested that the association between TCAs and seizures may be explained almost entirely by major depression.[25]

The underlying conditions may also require other kinds of treatment than antidepressants including antiepileptic and psychotropic drugs. However, when we excluded the users of these drugs from the analysis, the risk of epilepsy associated with SSRI use at time of TBI was virtually unchanged (S6 Table). Confounding from these drugs is thus unlikely to explain our findings.

To account for a potential bidirectional effect between epilepsy and depression,[26] we analyzed the data after stratifying in hospital admission with a depression diagnosis at any time prior to TIB (index date) to evaluate the effect of the underlying depression. A hospital admission with depression will most likely only capture the most severely affected individuals with depression but we had unfortunately no access to information on persons treated for depression in primary health care. Expectedly it will therefore be difficult to show any additional effect of SSRI on the risk of epilepsy after TBI and accordingly no additional effect could be identified among persons exposed to SSRI at time of TBI when compared to persons unexposed to SSRI at time of TBI among persons admitted to hospital with a diagnosis of depression (S7 Table).

Although the dataset was quite large, there were not enough cases to study individual SSRIs, and we did not have information on the individual drug dosages. These reservations constitutes limitations to the study results.

We studied the effects of use of SSRIs at time of TBI, but we did not include information on SSRI use after TBI. Because SSRIs are also used to treat sequelae from TBI such as anxiety and depression,[37] we would not be able to separate the risk of epilepsy associated with SSRIs from the risk of epilepsy associated with TBI. We therefore did not assess the risk of epilepsy in persons using SSRIs after TBI.

The strength of this study is that it is a nationwide cohort study with prospectively collected data and that all individuals who suffered a TBI and later reimbursed a prescription for SSRIs could be identified via national registers. The study is unlikely to suffer from selection bias because of the structure of the Danish healthcare system, in which all in-hospital treatment is provided free of charge and the costs of medication is progressively covered by the state from a minimum of 60% to a maximum of 100% for the costs above 3.955 Dkr/year (≈ 650 USD/year).[42]

The study is based on administrative data from registers and not on data collected as part of a specific clinical study which constitutes a limitation to the study. The study may thus be limited by the information on drug exposure (filling of prescriptions for SSRIs) and the diagnostic validity of the clinical diagnoses of TBI and epilepsy. We expect that most persons who reimburse SSRI prescriptions will also use the medication, but adherence with the medication may differ between persons with TBI and person without TBI. However, it is unlikely that the compliance with SSRI treatment is so much higher in the TBI group that it can explain the highly increased risk of epilepsy. Non-compliance with SSRI treatment would most likely lead to an underestimation of the association between SSRI use at time of TBI (index date) and the risk of epilepsy. We used ICD coding of TBI in order to determine TBI severity (mild, skull fracture and severe TBI), but we did not have access to clinical indicators for TBI severity including duration of unconsciousness and pre- and post-traumatic amnesia, and Glasgow Coma Scale. The definitions of “mild TBI” vary across studies,[43] however, the definitions used by most Danish hospitals are compatible with the definitions developed by the Mild Traumatic Brain Injury Committee of the Head Injury Interdisciplinary Special Interest Group of the American Congress of Rehabilitation Medicine.[21]

We previously assessed the positive predictive value of an epilepsy diagnosis in the Danish National Hospital Register and found that it is 81% (95% CI: 75–87%).[44] Therefore, some of the persons registered with epilepsy in this study may not actually have epilepsy. A person with TBI would probably be at higher suspicion for epilepsy which would lead to more intense follow up and thereby persons with TBI would more likely be diagnosed with epilepsy. This would tend to overestimate the risk of epilepsy after TBI, but we would not expect this additional risk to be influenced by the use of SSRI at time of diagnosis.

## Conclusion

Persons who used SSRI at the time of TBI had higher risk of epilepsy after TBI than one should expect from adding the individual risks associated with SSRIs and TBI. This association need not be causal, but could be due to confounding factors not accounted for in this study e.g. confounding from the an underlying disease (depression or anxiety). Also other factors that are important may include worse adherence to anti-seizure medications, increased alcohol use, and poorer sleep/self-care that have been clearly shown to be associated with depression/anxiety and are difficult to fully ascertain using administrative data. However, SSRI is common and even a small modifying effect on the risk of epilepsy after TBI could have important public health implications. Therefore, our study calls for further research into the potential role of SSRIs and underlying indications including depression on the risks of epilepsy after TBI.

## Supporting information

S1 Table Information on traumatic brain injury obtained from the National Patient Register. (DOCX)

S2 Table Information comorbidities obtained from the Danish National Patient Register and the Danish Psychiatric Central Research Register. (DOCX)

S3 Table Risk of epilepsy by use of Selective Serotonin Reuptake Inhibitors (SSRIs) at time of traumatic brain injury–restricted to persons born after 1. January 1977.
(DOCX)

S4 Table Risk of epilepsy by type of antidepressants use at time of traumatic brain injury. (DOCX)

S5 Table Risk of epilepsy by use of Selective Serotonin Reuptake Inhibitors (SSRIs) at time of traumatic brain injury–restricted to persons with traumatic brain injury between 1 Jan 1996 and 31 December 2009 who were then followed up to a maximum of 4 years after traumatic brain injury. (DOCX)

S6 Table Risk of epilepsy by use of Selective Serotonin Reuptake Inhibitors (SSRIs) at time of traumatic brain injury excluding persons with concomitant use of antiepileptic and psycholeptic drugs. (DOCX)

S7 Table Risk of epilepsy by hospital admission with a diagnosis of depression and use of Selective Serotonin Reuptake Inhibitors (SSRIs) at time of traumatic brain injury. (DOCX)
