# Supplementary material for: Selective serotonin reuptake inhibitors and risk of epilepsy after traumatic brain injury – A population based cohort study
Source: PLoS One. 2019 Jul 19;14(7):e0219137. doi: 10.1371/journal.pone.0219137 (PMC6641473; doi:10.1371/journal.pone.0219137)
Supplement: S1 Table — (DOCX) [file pone.0219137.s001.docx]

**S1 Table. Information on traumatic brain injury obtained from the Danish National Patient Register**

|  | | |
| --- | --- | --- |
|  | **ICD-8** | **ICD-10** |
| Mild brain injury (concussion) | 850.99 | S06.0 |
| Skull fracture | 800.99-801.09, 803.99 | S02-S02.1, S02.7, S02.9 |
| Severe brain injury | 851.29-854.99 | S06.1-S06.9 |
