# Supplementary material for: Selective serotonin reuptake inhibitors and risk of epilepsy after traumatic brain injury – A population based cohort study
Source: PLoS One. 2019 Jul 19;14(7):e0219137. doi: 10.1371/journal.pone.0219137 (PMC6641473; doi:10.1371/journal.pone.0219137)
Supplement: S2 Table — (DOCX) [file pone.0219137.s002.docx]

**S2 Table. Information comorbidities obtained from the Danish National Patient Register and the Danish Psychiatric Central Research Register**

|  | | |
| --- | --- | --- |
|  | **ICD-8** | **ICD-10** |
| ***Drug-related abuse*** |  |  |
| Opioids | 304.09, 304.19 | F11.0–F11.9 |
| Cannabinoids | 304.59 | F12.0–F12.9 |
| Sedatives/hypnotics | 304.29, 304.39 | F13.0–F13.9 |
| Cocaine | 304.49 | F14.0–F14.9 |
| Other stimulants | 304.69 | F15.0–15.9 |
| Hallucinogens | 304.79 | F16.0–F16.9 |
| Other and multiple drugs | 304.89, 304.99 | F18.0–F19.9 |
| ***Alcohol-related abuse*** |  |  |
| Alcohol psychosis and abuse syndrome | 291.09–291.99 | F10.0–F10.9 |
|  | 303.09–303.99 |  |
| Cirrhosis and steatosis of the liver | 571.09, 571.10, 571.19 | K70.0–K70.9 |
|  |  |  |
| ***Heart disease*** |  |  |
| Ischemic heart disease | 410–414 | I20–I25 |
| Atrial fibrillation or flutter | 427.93, 427.94 | I48 |
| Congestive heart failure | 427.09, 427.10, 427.11, 427.19, 428.99, 782.49 | I50; I11.0; I13.0; I13.2 |
| ***Other vascular diseases*** |  |  |
| Peripheral vascular disease | 440, 441, 442, 443, 444, 445, | I70; I71; I72; I73; I74; I77 |
| ***Autoimmune diseases*** |  |  |
| Pernicious Anemia | 281.00-09 | D51.0 |
| Autoimmune Haemolytic Anemia | 283.90-91 | D59.1 |
| Idiopathic Thrombocytopenic Purpura | 446.49 | D69.3 |
| Thyrotoxicosis | 242.00 | E05.0 |
| Autoimmune Thyroiditis | 245.03 | E06.3 |
| Type 1 Diabetes | 249.00-09 | E10 |
| Primary Adrenocortical Insufficiency | 255.10-19 | E27.1 |
| Multiple Sclerosis | 340.00-09 | G35 |
| Guillain Barre Syndrome | 354.00-09 | G61.0 |
| Iridocyclitis | 364.00-09 | H20 |
| Crohn’s Disease | 563.01 | K50 |
| Ulcerative Colitis | 563.19 | K51 |
| Autoimmune Hepatitis | 571.93 | K73 |
| Primary Biliary Cirrhosis | 571.90 | K74.3 |
| Celiac Disease | 269.00 | K90.0 |
| Pemphigus | 694.00-04, 694.08-09 | L10 |
| Pemphigoid | 694.05 | L12 |
| Psoriasis vulgaris | 696.09-10, 696.19 | L40.0-3, L40.5-9 |
| Alopecia Areata | 704.00 | L63 |
| Vitiligo | 709.01 | L80.9 |
| Seropositive Rheumatoid Arthritis | 712.19, 712.39, 712.59 | M05-M06 |
| Juvenile Arthritis | 712.09 | M08 |
| Wegener’s Granulomatosis | 446.29 | M31.3 |
| Dermatopolymyositis | 716.09-19 | M33 |
| Polymyalgia Rheumatica | 446.30-31, 446.39 | M31.5-6, M35.3 |
| Myasthenia Gravis | 733.09 | G70.0 |
| Scleroderma | 734.00-09 | M34 |
| Systemic Lupus Erythematosis | 734.19 | M32.1, M32.9 |
| Sjogren’s Syndrome | 734.90 | M35.0 |
|  |  |  |
| ***Infections*** |  |  |
| HIV | 079.83 | B20–24 |
