# Supplementary material for: Selective serotonin reuptake inhibitors and risk of epilepsy after traumatic brain injury – A population based cohort study
Source: PLoS One. 2019 Jul 19;14(7):e0219137. doi: 10.1371/journal.pone.0219137 (PMC6641473; doi:10.1371/journal.pone.0219137)
Supplement: S3 Table — January 1977. (DOCX) [file pone.0219137.s003.docx]

**S3 Table. Risk of epilepsy by use of Selective Serotonin Reuptake Inhibitors (SSRIs) at time of traumatic brain injury – restricted to persons born after 1. January 1977.**

|  |  |  |  |  | Risk of Epilepsy | | |
| --- | --- | --- | --- | --- | --- | --- | --- |
|  |  | Total (number) | Epilepsy (number) | Person Years | Crude  (95% CI) | Adjusted^a^  (95% CI) | Adjusted^a^  (95% CI) |
| Traumatic brain injury | SSRI | 1298 | 21 | 7265 | 5.66 (3.30;9.70) | 4.61 (2.63;8.09) | 1.88 (1.07; 3.31) |
|  | No SSRI | 101,348 | 1514 | 899,699 | 2.50 (2.36;2.64) | 2.45 (2.32;2.60) | 1.00 (ref) |
| No traumatic brain injury | SSRI | 5482 | 30 | 30,465 | 1.86 (1.26;2.75) | 1.65 (1.10;2.47) | 1.65 (1.10;2.47) |
|  | No SSRI | 1,020,978 | 6110 | 9,077,039 | 1.00 (ref) | 1.00 (ref) | 1.00 (ref) |

^a^Adjusted for civil status, income, medical and neurological comorbidities, schizophrenia, bipolar affective disorder, and substance abuse.
