# Supplementary material for: Selective serotonin reuptake inhibitors and risk of epilepsy after traumatic brain injury – A population based cohort study
Source: PLoS One. 2019 Jul 19;14(7):e0219137. doi: 10.1371/journal.pone.0219137 (PMC6641473; doi:10.1371/journal.pone.0219137)
Supplement: S4 Table — (DOCX) [file pone.0219137.s004.docx]

**S4 Table. Risk of epilepsy by type of antidepressants use at time of traumatic brain injury**

| Traumatic brain injury | Antidepressant use | Total | Epilepsy | Person Years | Risk of Epilepsy | |  |
| --- | --- | --- | --- | --- | --- | --- | --- |
|  |  | number) | (number) |  | Crude  (95% CI) | Adjusted^a^  (95% CI) |  |
| Traumatic brain injury | SSRIs (monotherapy) | 10,176 | 394 | 54,911 | 9.13 (7.94;10.50) | 5.62 (4.84;6.52) | 1.74 (1.50; 2.00) |
|  | No antidepressants | 189,963 | 4246 | 1,616,331 | 3.64 (3.51;3.77) | 3.22 (3.10;3.34) | 1.00 (ref) |
| No traumatic brain injury | SSRIs (monotherapy) | 41,572 | 427 | 229,107 | 1.83 (1.64;2.03) | 1.35 (1.21;1.51) | 1.35 (1.21;1.51) |
|  | No antidepressants | 1,986,257 | 13,101 | 17,495,090 | 1.00 (ref) | 1.00 (ref) | 1.00 (ref) |
| Traumatic brain injury | TCAs (monotherapy) | 1297 | 41 | 7719 | 4.76 (3.25;6.97) | 3.00 (2.01;4.47) | 0.93 (0.62; 1.39) |
|  | No antidepressants | 189,963 | 4246 | 1,616,331 | 3.64 (3.51;3.77) | 3.22 (3.10;3.34) | 1.00 (ref) |
| No traumatic brain injury | TCAs (monotherapy) | 8,414 | 136 | 56,674 | 1.96 (1.63;2.36) | 1.64 (1.35;1.98) | 1.64 (1.35;1.98) |
|  | No antidepressants | 1,986,257 | 13,101 | 17,495,090 | 1.00 (ref) | 1.00 (ref) | 1.00 (ref) |
| Traumatic brain injury | Other ADs (monotherapy) | 2730 | 110 | 12,462 | 12.24 (9.22;16.24) | 8.12 (6.01;10.98) | 2.52 (1.86; 3.42) |
|  | No antidepressants | 189,963 | 4246 | 1,616,331 | 3.64 (3.51;3.77) | 3.22 (3.10;3.34) | 1.00 (ref) |
| No traumatic brain injury | Other ADs (monotherapy) | 14,244 | 156 | 68,761 | 2.29 (1.92;2.73) | 1.78 (1.48;2.13) | 1.78 (1.48;2.13) |
|  | No antidepressants | 1,986,257 | 13,101 | 17,495,090 | 1.00 (ref) | 1.00 (ref) | 1.00 (ref) |
| Traumatic brain injury | MAOIs (or polyfarmaci) | 1549 | 67 | 6903 | 11.48 (8.06;16.35) | 7.15 (4.91;10.42) | 2.22 (1.52; 3.24) |
|  | No antidepressants | 189,963 | 4246 | 1,616,331 | 3.64 (3.51;3.77) | 3.22 (3.10;3.34) | 1.00 (ref) |
| No traumatic brain injury | MAOIs (or polyfarmaci) | 6663 | 75 | 32,079 | 2.01 (1.56;2.58) | 1.31 (1.01;1.71) | 1.31 (1.01;1.71) |
|  | No antidepressants | 1,986,257 | 13,101 | 17,495,090 | 1.00 (ref) | 1.00 (ref) | 1.00 (ref) |

SSRIs: Selective serotonin reuptake inhibitors, MAOIs: Monoamine oxidase inhibitors, ADs: Other antidepressants, TCAs: Tricyclic Antidepressants

^a^Adjusted for civil status, income, medical and neurological comorbidities, schizophrenia, bipolar affective disorder, and substance abuse.
