# Supplementary material for: Selective serotonin reuptake inhibitors and risk of epilepsy after traumatic brain injury – A population based cohort study
Source: PLoS One. 2019 Jul 19;14(7):e0219137. doi: 10.1371/journal.pone.0219137 (PMC6641473; doi:10.1371/journal.pone.0219137)
Supplement: S5 Table — (DOCX) [file pone.0219137.s005.docx]

**S5 Table. Risk of epilepsy by use of Selective Serotonin Reuptake Inhibitors (SSRIs) at time of traumatic brain injury – restricted to persons with traumatic brain injury between 1 Jan 1996 and 31 December 2009 who were then followed up to a maximum of 4 years after traumatic brain injury.**

|  |  |  |  |  | Risk of Epilepsy | |
| --- | --- | --- | --- | --- | --- | --- |
|  |  | Total (number) | Epilepsy (number) | Person Years | Adjusted^a^  (95% CI) | Adjusted^a^  (95% CI) |
| Traumatic brain injury | SSRI | 8744 | 279 | 27,285 | 7.43 (6.13;9.00) | 1.65 (1.35; 2.01) |
|  | No SSRI | 157,979 | 2432 | 589,695 | 4.51 (4.28;5.75) | 1.00 (ref) |
| No traumatic brain injury | SSRI | 35,080 | 247 | 117,014 | 1.32 (1.14;1.53) | 1.32 (1.14;1.53) |
|  | No SSRI | 1,632,150 | 5101 | 6,292,584 | 1.00 (ref) | 1.00 (ref) |

^a^ Adjusted for civil status, income, medical and neurological comorbidities, schizophrenia, bipolar affective disorder, and substance abuse.
