# Supplementary material for: Selective serotonin reuptake inhibitors and risk of epilepsy after traumatic brain injury – A population based cohort study
Source: PLoS One. 2019 Jul 19;14(7):e0219137. doi: 10.1371/journal.pone.0219137 (PMC6641473; doi:10.1371/journal.pone.0219137)
Supplement: S6 Table — (DOCX) [file pone.0219137.s006.docx]

**S6 Table. Risk of epilepsy by use of Selective Serotonin Reuptake Inhibitors (SSRIs) at time of traumatic brain injury excluding persons with concomitant use of antiepileptic and psycholeptic drugs**.

| **Excluding persons using antiepileptic drugs**  **(ATC code: N03A)** | | Total (number) | Epilepsy (number) | Person Years | Crude  (95% CI) | Adjusted^a^  (95% CI) | Adjusted^a^  (95% CI) | |
| --- | --- | --- | --- | --- | --- | --- | --- | --- |
| Traumatic brain injury | SSRI | 10,877 | 402 | 57,587 | 9.24 (8.04; 10.63) | 5.80 (5.00; 6.74) | 1.76 (1.51; 2.06) | |
|  | No SSRI | 192,131 | 4,213 | 1,627,577 | 3.71 (3.58; 3.85) | 3.29 (3.17; 3.41) | 1.00 (ref) | |
| No traumatic brain injury | SSRI | 45,510 | 419 | 247,904 | 1.67 (1.50; 1.86) | 1.25 (1.12; 1.40) | 1.25 (1.12; 1.40) | |
|  | No SSRI | 2,000,830 | 12,724 | 17,578,749 | 1.00 (ref) | 1.00 (ref) | 1.00 (ref) | |
|  |  |  |  |  |  |  |  | |
| **Excluding persons using anxiolytic drugs**  **(ATC code: N05B)** | | Total (number) | Epilepsy (number) | Person Years | Crude  (95% CI) | Adjusted^a^  (95% CI) |  | |
| Traumatic brain injury | SSRI | 8,832 | 312 | 43,864 | 8.89 (7.59; 10.42) | 5.73 (4.84; 6.79) | 1.79 (1.51; 2.13) | |
|  | No SSRI | 186,976 | 4,025 | 1,588,902 | 3.56 (3.43; 3.69) | 3.19 (3.08; 3.32) | 1.00 (ref) | |
| No traumatic brain injury | SSRI | 39,288 | 372 | 206,850 | 1.76 (1.57; 1.98) | 1.29 (1.15; 1.46) | 1.29 (1.15; 1.46) | |
|  | No SSRI | 1,971,062 | 12,820 | 17,344,703 | 1.00 (ref) | 1.00 (ref) | 1.00 (ref) | |
|  |  |  |  |  |  |  |  | |
| **Excluding persons using hypnotic and sedative drugs**  **(ATC code: N05C)** | | Total (number) | Epilepsy (number) | Person Years | Crude  (95% CI) | Adjusted^a^  (95% CI) |  | |
| Traumatic brain injury | SSRI | 9,010 | 330 | 47,422 | 9.14 (7.82; 10.68) | 5.93 (5.03; 7.00) | 1.86 (1.56; 2.21) | |
|  | No SSRI | 186,665 | 4,058 | 1,594,393 | 3.56 (3.43; 3.69) | 3.18 (3.06; 3.30) | 1.00 (ref) | |
| No traumatic brain injury | SSRI | 39,147 | 386 | 212,394 | 1.77 (1.58; 1.98) | 1.30 (1.16; 1.47) | 1.30 (1.16; 1.47) | |
|  | No SSRI | 1,959,173 | 12,830 | 17,308,873 | 1.00 (ref) | 1.00 (ref) | 1.00 (ref) | |
|  |  |  |  |  |  |  |  | |
| **Excluding persons using antipsychotic drugs**  **(ATC code: N05A)** | | Total (number) | Epilepsy (number) | Person Years | Crude  (95% CI) | Adjusted^a^  (95% CI) |  | |
| Traumatic brain injury | SSRI | 10,069 | 363 | 53,281 | 8.54 (7.39; 9.87) | 5.40 (4.63; 6.30) | 1.68 (1.44; 1.97) | |
|  | No SSRI | 191,017 | 4,243 | 1,620,973 | 3.63 (3.50; 3.76) | 3.21 (3.09; 3.33) | 1.00 (ref) | |
| No traumatic brain injury | SSRI | 41,799 | 409 | 228,032 | 1.70 (1.52; 1.89) | 1.27 (1.14; 1.43) | 1.27 (1.14; 1.43) | |
|  | No SSRI | 1,991,693 | 13,116 | 17,515,103 | 1.00 (ref) | 1.00 (ref) | | 1.00 (ref) |

^a^Adjusted for civil status, income, medical and neurological comorbidities, schizophrenia, bipolar affective disorder, and substance abuse.
