# Supplementary material for: Selective serotonin reuptake inhibitors and risk of epilepsy after traumatic brain injury – A population based cohort study
Source: PLoS One. 2019 Jul 19;14(7):e0219137. doi: 10.1371/journal.pone.0219137 (PMC6641473; doi:10.1371/journal.pone.0219137)
Supplement: S7 Table — (DOCX) [file pone.0219137.s007.docx]

**S7 Table. Risk of epilepsy by hospital admission with a diagnosis of depression and use of Selective Serotonin Reuptake Inhibitors (SSRIs) at time of traumatic brain injury.**

| Traumatic brain injury | Depression | SSRI use | Total  (number) | Epilepsy  (number) | Person Years | Risk of Epilepsy | | |
| --- | --- | --- | --- | --- | --- | --- | --- | --- |
|  |  |  |  |  |  | Crude  (95% CI) | Adjusted*  (95% CI) | Adjusted*  (95% CI) |
| Traumatic brain injury | Depression | SSRI | 1,773 | 74 | 9,890 | 9.53 (6.91;13.16) | 4.84 (3.41;6.86) | 0.84 (0.55; 1.28) |
|  |  | No SSRI | 3,704 | 176 | 21,631 | 10.88 (8.75;13.54) | 5.76 (4.56;7.28) | 1.00 (ref) |
|  | No depression | SSRI | 9,811 | 379 | 51,209 | 9.27 (8.03;10.69) | 5.88 (5.05;6.84) | 1.84 (1.57; 2.15) |
|  |  | No SSRI | 190,427 | 4229 | 1,615,597 | 3.60 (3.48;3.73) | 3.20 (3.09;3.32) | 1.00 (ref) |
| No traumatic brain injury | Depression | SSRI | 6,616 | 68 | 35,052 | 2.08 (1.60;2.70) | 1.25 (0.94;1.64) | 0.94 (0.69; 1.29) |
|  |  | No SSRI | 18,000 | 207 | 111,774 | 1.88 (1.61;2.18) | 1.32 (1.13;1.54) | 1.00 (ref) |
|  | No depression | SSRI | 40,908 | 427 | 221,670 | 1.80 (1.62;2.00) | 1.34 (1.20;1.50) | 1.34 (1.20;1.50) |
|  |  | No SSRI | 1,991,626 | 13,193 | 17,513,215 | 1.00 (ref) | 1.00 (ref) | 1.00 (ref) |

* Adjusted for civil status, medical and neurological comorbidities, schizophrenia, bipolar affective disorder, and substance abuse.
